# Effects of Alternative Administration Programs of a Synbiotic Supplement on Broiler Performance, Foot Pad Dermatitis, Caecal Microbiota, and Blood Metabolites

**DOI:** 10.3390/ani10030522

**Published:** 2020-03-20

**Authors:** Giorgio Brugaletta, Alessandra De Cesare, Marco Zampiga, Luca Laghi, Chiara Oliveri, Chenglin Zhu, Gerardo Manfreda, Basharat Syed, Luis Valenzuela, Federico Sirri

**Affiliations:** 1 Department of Agricultural and Food Sciences, Alma Mater Studiorum–University of Bologna, Ozzano dell’Emilia, 40064 Bologna, Italy; giorgio.brugaletta2@unibo.it (G.B.); marco.zampiga2@unibo.it (M.Z.); l.laghi@unibo.it (L.L.); chiara.oliveri5@unibo.it (C.O.); chenglin.zhu2@unibo.it (C.Z.); gerardo.manfreda@unibo.it (G.M.); federico.sirri@unibo.it (F.S.); 2 Department of Veterinary Medical Sciences, Alma Mater Studiorum–University of Bologna, Ozzano dell’Emilia, 40064 Bologna, Italy; 3 Biomin Holding, 3131 Getzersdorf, Austria; basharat.syed@biomin.net (B.S.); luis.valenzuela@biomin.net (L.V.)

**Keywords:** poultry gut health, broiler, synbiotic supplement, administration program, performance, feed conversion ratio, foot pad dermatitis, caecal microbiota, plasma metabolite

## Abstract

**Simple Summary:**

Banning antibiotics as feed additives has brought out the impelling necessity to develop reliable and cost-effective alternatives to improve livestock performance without undermining public health. Synbiotic supplements enhance poultry gut health, which in turn affects productivity and general well-being. Synbiotics have been traditionally supplemented in-feed or via drinking water. However, in-ovo injection and spraying aqueous suspensions on feathering of newly hatched chicks were proposed to allow desirable strains to early colonize the gut and hinder harmful microorganisms more effectively. The aim of this study was to investigate the effects of alternative administration programs of a synbiotic on broiler’s productive performance, foot pad dermatitis, caecal microbiota, and plasma metabolites. Favorable effects on feed efficiency and foot pad conditions were observed when the synbiotic was -delivered as gel droplets at the hatchery combined to in-feed supplementation during the entire growing cycle. Such improvements can be ascribed to the potential modulatory effect of the synbiotic towards gastro-intestinal microbial community. Significant differences of plasma ascorbic acid and propylene-glycol levels were also observed in treated animals.

**Abstract:**

This research investigated the effects of different synbiotic administration programs on broiler productive performance and foot pad dermatitis (FPD). Molecular insights on caecal microbiota and plasma metabolomics were also performed. - A total of 1000 one-day-old male chicks were grouped by the synbiotic treatment. The synbiotic was either sprayed as gel droplets onto newly hatched chicks at the hatchery (100 g/10,000 birds) or supplemented in-feed during the entire rearing period (1000, 500, and 250 g/ton according to feeding phase), or both. Only the treatments’ combination produced significant results in comparison with the control group (untreated), improving feed conversion ratio from 14 to 29 d and in the overall period of the trial (1.570 vs. 1.509 and 1.643 vs. 1.596, respectively; *p* < 0.05) while lowering FPD occurrence at slaughter (17% vs. 5%; *p* < 0.05). These findings can be related to significant variations of caecal microbiota, like higher Firmicutes to Bacteroidetes ratio (with favorable implications for host’s energy-harvesting potential from the diet) and more beneficial microbial consortium presumably sustaining eubiosis. Overall, these results indicate that administering synbiotics through gel droplets at the hatchery combined to in-feed supplementation for the whole growing cycle positively affects broiler feed efficiency and welfare.

## 1. Introduction

During the last three decades, the way of considering the gastrointestinal tract (GIT) of food-producing animals has been revolutionised. This new point of view made us aware of the real complexity of such anatomical system fulfilling digestive, absorptive, metabolic, immunological, and endocrinological roles [1]. That is why the locution “gut health” entered collective consciousness of livestock industry and researchers [2], becoming a new paradigm in animal science [3]. The features of healthy GIT are hard to explain, especially because they depend on a multitude of covariates related to diet, digestion and absorption processes, plasticity and resilience of the gastroenteric immunity, morpho-physiological integrity of the gastrointestinal (GI) barrier, and microbiota stability. Nonetheless, Celi and colleagues [4] were able to thoroughly describe the gut health concept emphasizing the importance of microbiome-gut balanced symbiotic relationship and the relevant influence of intestinal functionality towards animal health and productiveness.

Antibiotics have been the faithful ally of breeders in search of GI equilibrium of farm animals. The discoveries of Moore et al. [5] and Jukes et al. [6] officially inaugurated the antibiotic growth-promoters (AGPs) era and, over the last decades, several studies have confirmed beneficial effects of subtherapeutic doses of antimicrobials administered to livestock. Nevertheless, an immoderate use of these molecules in farms has contributed to the spreading of antimicrobial resistance (AMR), which is a phenomenon threatening human and animal health, along with the environment [7,8]. Animal husbandry dependence on AGPs has been criticized by civil society in many countries and, eventually, in some jurisdictions the policymaker legislated for their limitation or prohibition in food-producing animals. For example, via the Reg. EC 1831/2003 [9], the EU imposed the total ban (in force since 1 January 2006) of growth-promoting antibiotics. The European model has been followed by South Korea, whereas China has issued an action plan encouraging AGPs suspension and Brazil has already imposed severe directive on their use in agriculture [10,11,12]. 

However, there are numerous drawbacks in breeding livestock, especially poultry, without AGPs. Since gut health has wide consequences for poultry systemic health, animal welfare, flock production and efficiency, as well as food safety and environmental impact [1], banning antibiotics as feed additives has created challenges for poultry producers, bringing out the impelling necessity to develop reliable and cost-effective alternatives in order to improve animals’ performance without undermining public health [13].

Probiotics and prebiotics ensure diversity and stability of the GI microbial community, as well as positive interactions with host’s gastroenteric epithelium and immune system [14]. Moreover, several studies and reviews on poultry have reported that the simultaneous use of probiotics and prebiotics blended in an unique supplement (i.e., a synbiotic) is more efficacious than administering single preparations or, in some cases, even comparable to antibiotic treatments [14,15,16,17,18]. 

Probiotic preparations have been traditionally supplemented in-feed or via drinking water during broiler growing cycles. This makes the USDA denomination of such additives, namely “direct-fed microbials” [19], easily understandable. However, in-ovo injection [20] and spraying solutions [21] on the feathering of newly hatched chicks were proposed to foster an early GI colonization by probiotic strains and to enforce competitive exclusion (CE) against harmful microorganisms. Such early administration techniques have led to positive results in terms of chicken welfare and performance [22,23,24]. Moreover, it was recently demonstrated that providing a probiotic to hatchlings positively and permanently changes their GI microbiota while significantly enhancing the body weight during growth [25].

In such context, this research investigated the effects produced by alternative administration programs of a synbiotic on broiler’s performances and foot pad dermatitis (FPD). The additive was either sprayed, in an unusual form (i.e., gel droplets), on the feathering of newly hatched chicks or added as microcapsules to the feed during the entire rearing period, or both. Molecular insights on caecal microbiota and plasma metabolites were also carried out 

## 2. Materials and Methods 

### 2.1. Animals and Management

A total of 1000 one-day-old male Ross 308 chicks, obtained from the same breeder flock and hatching session, were used. At the hatchery all chicks were vaccinated against infectious bronchitis virus, Marek’s disease virus, Newcastle and Gumboro diseases, and coccidiosis.

At placement in an experimental poultry house, chicks were divided into 4 groups of 10 replications each (25 chicks/replication). The replications were distributed, in randomized blocks in order to reduce any environmental effect, in 40 pens (2.5 m^2^/pen) at stocking density of 25 birds/pen.

Wood shavings (3–4 kg/m^2^) were employed as litter material. Pens were provided with pan feeders, ensuring at least 2 cm/bird of front space, and an independent drinking system with at least 1 nipple/5 birds. Feeders were of identical manufacture, type, size, and colour. Each pen was equipped with an individual bin, clearly labelled as reservoir for the experimental feed. Daily, experimental diets were manually transferred from bins to feeders. Feed and water were provided ad libitum. Any diet change was uniformly performed for all animals. At each diet switch, feeders were emptied, and residuals were weighed. Afterwards, feeders were filled according to the feeding program, which encompassed 3 phases: starter (0–14 d), grower (15–28 d), and finisher (29–42 d).

A photoperiod of 23L:1D of artificial light was adopted during the first 7 d and the last 3 d of the trial, whereas 18L:6D was used for the remaining days. Environmental temperature was settled according to birds’ age, following the management guide provided by the breeding company. Birds were handled, raised, and processed in compliance with the European legislation [26,27,28]. Inspections were carried out twice a day to monitor general flock condition, temperature, lighting, water, feed, litter, and mortality.

The trial lasted 42 days when birds reached the slaughter weight of about 3 kg. This experiment was approved by the Ethical Committee of the University of Bologna (ID: 1049/2019).

### 2.2. Experimental Diets and Synbiotic Treatments

The experimental groups were labelled from A to D according to post-hatch and dietary treatments with the synbiotic. Group A represented the control which received no synbiotic supplementation neither at the hatchery nor in-feed; group B was provided with the synbiotic in-feed throughout the growing cycle; group C received the post-hatch variant of the synbiotic and the basal diet during the rearing period; group D was treated with the synbiotic both at the hatchery and through the feed. 

**Figure 1:**
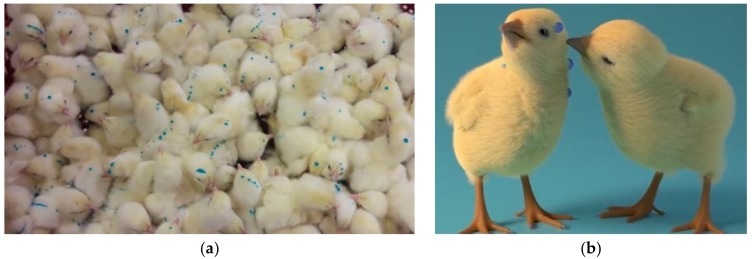
(**a**) Post-hatch administration of PoultryStar^®^ Hatchery^EU^ (source: Biomin); (**b**) Chicks’ mutual grooming triggered by PoultryStar^®^ Hatchery^EU^.

According to manufacturer instructions, at the hatchery the synbiotic PoultryStar^®^ Hatchery^EU^ (Biomin, Austria) was provided, at concentration of 100 g/10,000 birds, through spraying blue gel droplets on the feathering, which were able to trigger preening reflex in fledglings (Figure 1). The synbiotic preparation was constituted by *Bifidobacterium animalis* ssp. *animalis*, *Lactobacillus salivarius* ssp. s*alivarius*, *Enterococcus faecium* (3:1:6 ratio, 1 × 10^13^ CFU/kg), and FOS as prebiotic element. On average, each treated chick ingested 10^8^ CFU of probiotic bacteria. 

The microencapsulated version of the synbiotic intended for feed application (PoultryStar^®^ me^EU^, Biomin) was provided at inclusion levels of 1000, 500, and 250 g/ton during starter, grower, and finisher phase, respectively. Microbial and prebiotic compositions of PoultryStar^®^ me^EU^ were equal to that of PoultryStar^®^ Hatchery^EU^. However, the feed additive had a bacterial concentration of 2 × 10^11^ CFU/kg. Therefore, considering the total feed intake of roughly 5 kg in 42 days and the inclusion levels previously listed, synbiotic-fed birds approximately got 4.5 × 10^8^ CFU of probiotic microorganisms during the rearing period.

Feed was administered in mash form and, according to the feeding scheme, the commercial corn-wheat-soybean basal diet varied as reported in Table 1. 

### 2.3. Productive Performance and Evaluation of FPD

On a pen basis number and weight of birds were recorded at housing (0 d), at each diet switch (14 and 28 d), and at slaughter (42 d). Feed intake was recorded at the end of each feeding phase (14, 28, and 42 d). Mortality was recorded daily and dead birds were weighed and recorded to calculate mortality percentage and to correct productive performance results. Body weight (BW), daily weight gain (DWG), daily feed intake (DFI), feed conversion ratio (FCR), and cumulative FCR were determined for each feeding phase and for the overall rearing period.

At 42 d all birds were processed in a commercial plant and slaughtered in compliance with the European legislation [27] using water-bath electrical stunning (200–220 mA, 1500 Hz). Birds and carcasses belonging to different experimental groups were clearly identified and separately kept throughout the processing phases. 

Following the classification method proposed by Ekstrand and collegues [29], incidence and severity of FPD were macroscopically evaluated on all birds (1 foot/bird) using a 3-point scale: score 0 = no lesion; score 1 = mild lesions (≤ 0.8 cm); score 2 = severe lesions (> 0.8 cm).

### 2.4. Blood and Caecal Content Collections

At slaughter (42 d) 1 bird/replication (i.e., 10 birds/group) was selected according to similar BW, clearly labelled, and subjected to blood withdrawal. Blood was obtained from the wing vein, collected into 4 mL lithium-heparin vials and centrifuged (4000× *g* for 15 min) to obtain plasma, which was transferred into 1.5 mL vials and stored at −80 °C until metabolomic analysis. From the same birds, the entire GIT was dissected out and the caecal content was collected into 15 mL sterile plastic tubes. Caecal samples were stored at −80 °C until DNA extraction.

### 2.5. Plasma Metabolomics Analyses

Plasma samples were prepared for proton nuclear magnetic resonance (^1^H-NMR) analysis by centrifuging 650 μL of each sample for 15 min at 15,000 r/min (18,630× *g*) and 4 °C. 500 μl of supernatant were added to 100 μL of a D_2_O solution of 2,2,3,3-D4-3-(trimethylsilyl)-propionic acid sodium salt 10 mmol/L, used as NMR chemical-shift reference, buffered at pH 7.00 by means of 1 mol/L phosphate buffer. Finally, each sample was centrifuged again at the above conditions. ^1^H-NMR spectra were recorded at 298 K with an AVANCE™ III spectrometer (Bruker, Milan, Italy) operating at a frequency of 600.13 MHz.

Following Ventrella et al. [30], signals from broad resonances originating from large molecules were suppressed by a CPMG-filter composed by 400 echoes with a τ of 400 μs and a 180° pulse of 24 μs, for a total filter of 330 ms. The water residual signal was suppressed by means of presaturation. This was done by employing the cpmgpr1d sequence, part of the standard pulse sequence library. Each spectrum was acquired by summing up 256 transients using 32,000 data points over a 7184 Hz spectral window, with an acquisition time of 2.28 s. In order to apply ^1^H-NMR as a quantitative technique [31], the recycle delay was set to 5 s, keeping into consideration the relaxation time of the protons under investigation. 

^1^H-NMR spectra were baseline-adjusted by means of the peak detection according to the “rolling ball” principle [32] implemented in the baseline R package [33]. In order to make points pertaining to the baseline randomly spread around zero, a linear correction was then applied to each spectrum. Differences in water content among samples were taken into consideration by probabilistic quotient normalization [34] applied to the entire spectra array.

Signals were assigned by comparing their chemical shift and multiplicity with the Human Metabolome Database [35] and Chenomx software library (Chenomx Inc., Edmonton, Canada, ver. 10). This was done by taking advantage of the “autofit” utility of Chenomx software (Chenomx Inc., ver. 8.3).

### 2.6. DNA Extraction Protocol

The DNA was extracted from each caecal sample using a bead-beating procedure [36]. Briefly, 0.25 g of caecal content were suspended in 1 mL lysis buffer (500 mM NaCl, 50 mM Tris-Cl, pH 8.0, 50 mM EDTA, 4% SDS) with MagNA Lyser Green Beads (Roche, Milan, Italy) and homogenized on the MagNA Lyser (Roche) for 25 s at 6.500 rpm. Samples were then heated at 70 °C for 15 min, followed by centrifugation to separate the DNA from bacterial cellular debris. This process was repeated with a second 300 μL aliquot of lysis buffer. Samples were then subjected to 10 M v/v ammonium acetate (Sigma, Milan, Italy) precipitation, followed by isopropanol (Sigma) precipitation, 70% ethanol (Carlo Erba, Milan, Italy) washing and suspension in 100 μL 1X Tris-EDTA (Sigma). All samples were treated with DNase-free RNase (Roche) and incubated overnight at 4 °C, before being processed through the QIAmp^®^ DNA Stool Mini Kit (Qiagen, Milan, Italy) according to manufacturer’s directions with some modifications. Lastly, DNA quantity and quality were assessed on a BioSpectrometer^®^ (Eppendorf, Milan, Italy).

### 2.7. 16S rRNA Amplicon Sequencing

Libraries were prepared following the 16S Metagenomic Sequencing Library Preparation protocol (Illumina, San Diego, CA, USA), amplifying V3 and V4 hypervariable regions of the 16S rRNA gene in order to obtain a single amplicon of approximately 460 bp. Sequencing was performed in paired-end employing MiSeq System (Illumina) with MiSeq Reagent kit v2 500 cycles (Illumina), characterised by a maximum output of 8.5 Gb.

### 2.8. Statistical Analysis of Productive Performance and FPD

Productive performance data of the 4 experimental groups were analysed applying one-way ANOVA with significance level of 5%, followed by Tukey’s post-hoc test. Pen was considered as the experimental unit for productive performance analyses. Before carrying out such analyses, mortality data were submitted to arcsine transformation. Occurrence and severity of FPD at 42 d were examined using the Chi-square test involving all the experimental groups and considering each bird as the experimental unit.

Pairwise comparisons between the control and each treated group were also performed by applying the Student’s *t*-test on productive performance parameters and the Chi-square test on FPD. 

### 2.9. Statistical Analysis of Caecal Bacteria Relative Abundances and of Plasma Metabolomics

The relative abundances of identified caecal bacteria were analysed, at each taxonomic level, with mg-RAST [37] by querying three databases, namely SILVA [38], Greengenes [39] and RDP [40]. The data sets were downloaded from mg-RAST and subsequently analysed by means of STAMP [41]. SILVA’s data were used for graphic representations.

Since groups A and D showed significant differences in terms of performance and FPD, we tried to elucidate the molecular mechanisms of the synbiotic treatments. Plasma metabolomes of A and D were analysed with the Student’s *t*-test, and taxonomic data of their caecal samples were compared by means of the Student’s *t*-test and the Welch’s *t*-test. We focused on data retrieved from SILVA to make graphic elaborations.

## 3. Results

### 3.1. Productive Performance and FPD 

In Table 2 productive performance of each feeding phase and for the entire period are given. Chicks showed comparable weights among groups. Taking into consideration all experimental groups, no significant variation in terms of productive performance was detected during each feeding phase as well as in the overall period of the trial -. 

However, the Student’s *t*-test used to test differences between the control (A) and each experimental group (B, C and D) highlighted significant outcomes only for group A vs. D one (Table 3). At the end of starter feeding phase, D exhibited lower mortality rate (%) compared to A (0.00 vs. 2.34, respectively; *p* < 0.01), whereas the synbiotic treatments unaffected other performance items. Dissimilarities enlarged during the second feeding phase, which ended at 29 d of life. At that time, A showed a lower feed efficiency (1.570 vs. 1.509, for A and D respectively; *p* < 0.05) and worse cumulative FCR from 0 to 29 d (1.506 vs. 1.467; *p* = 0.06).The tendency of D to express better productive efficiency was confirmed during the last period of the trial (i.e., 30–42 d), when treated chickens showed lower FCR than their untreated counterparts (1.753 vs. 1.809; *p* = 0.07). Considering the whole experiment duration (i.e., 0–42 d), the double synbiotic administration significantly improved FCR (1.643 vs. 1.596, for A and D respectively; *p* = 0.01).

Treating C and D fledglings with the synbiotic preparation at the hatchery tended to lessen occurrence and severity of FPD (*p* = 0.07). FPD reduction appeared greater for D birds (Table 4). Such trend becomes significant when A is analysed with D. Indeed, incidence and severity of FPD were markedly fewer in animals getting the supplement both post-hatch and during the growing cycle (Table 5).

### 3.2. Plasma Metabolome

The concentration of 61 plasma molecules for each replication of the 4 experimental groups is given in Table S1: Plasma metabolomics profiles according to replication.

Student’s *t-*test revealed that ascorbate level was significantly lower (0.019 vs. 0.024 mmol/L; *p* < 0.05) and propylene-glycol concentration significantly higher (0.007 vs. 0.001 mmol/L; *p* < 0.05) in D than A.

### 3.3. Caecal Microbiota 

**Figure 2:**
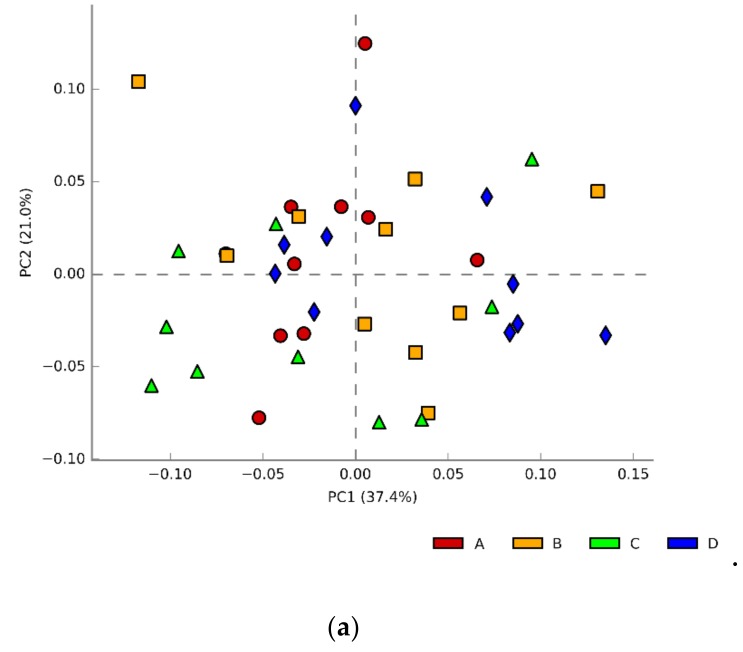

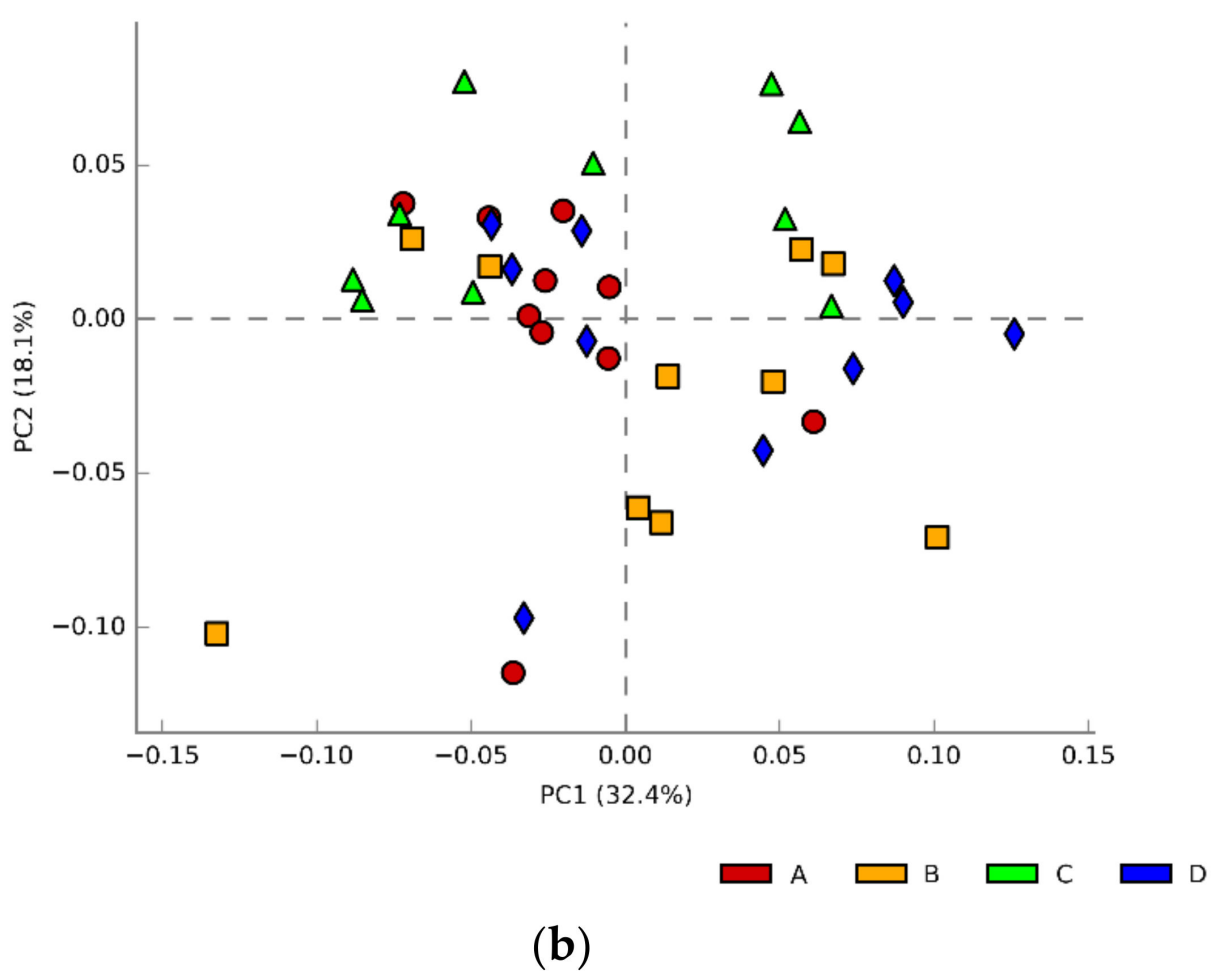
(**a**) Comparison of caecal microbial communities of all experimental groups (family level); (**b**) comparison of caecal microbial communities of all experimental groups (genus level).

Considering all the experimental groups, PCA analysis on data retrieved from SILVA shows no clear differentiation of caecal microbiotas at family and genus levels (Figure 2). 

Significant results of Student’s *t*-test and Welch’s *t*-test involving A and D and based on SILVA, Greengenes, and RDP data are given in Table S2: Mean relative frequency of abundance (%) of phyla, classes, orders, genera, and species of caecal bacteria in 42-day old broilers belonging to groups A and D.

**Figure 3:**
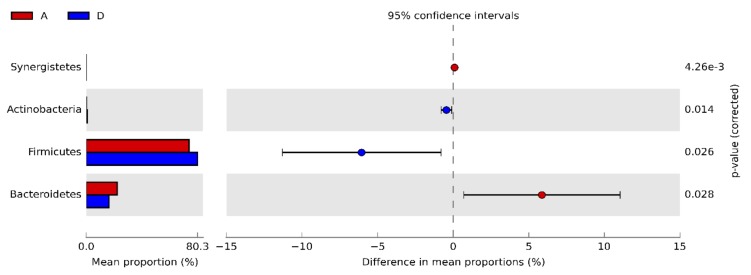
Bacterial phyla resulting significantly different in synbiotic-fed broilers (group D) in comparison to untreated birds (group A) at 42 d.

At phylum level, Actinobacteria and Firmicutes were significantly higher in D than A (0.22% vs. 0.67% and 74.20% vs. 80.27%, respectively), whereas Bacteroidetes and Synergistetes were significantly lower (16.55% vs. 22.42% and 0.08% vs. 0.17%, respectively) (Figure 3).

**Figure 4:**
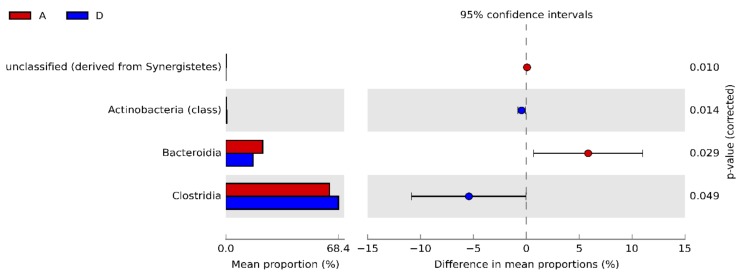
Bacterial classes resulting significantly different in synbiotic-fed broilers (group D) in comparison to untreated birds (group A) at 42 d.

At class level, Bacteroidia was significantly higher in A compared to D (22.35% vs. 16.51%, respectively), whereas Clostridia and Actinobacteria showed higher abundance in the synbiotic-fed group (62.95% vs. 68.38% and 0.22% vs. 0.67%, respectively) (Figure 4). 

At order level, Actinomycetales and Clostridiales were more abundant in D than A (0.42% vs. 0.11% and 68.29% vs. 62.65%, respectively). Furthermore, within Bacteroidales order three genera, namely *Bacteroides*, *Parabacteroides*, and *Prevotella* were significantly less abundant in D than A (15.13% vs. 10.54%, 0.01% vs. 0.004%, and 0.004% vs. 0.002%, respectively), whereas *Collinsella* was significantly higher (0.01% vs. 0.003%).

Focusing on bacterial species, the double supplementation caused the following variations: *Bifidobacterium longum*, *Collinsella intestinalis*, *Lactobacillus panis*, *Lactobacillus reuteri*, uncultured *Streptococcus sp.*, *Clostridium cocleatum*, *Clostridium difficile*, *Clostridium innocuum*,* Eubacterium ramulus*,* Peptoniphilus asaccharolyticus*, *Ruminococcus obeum*, *Blautia producta*, *Blautia sp*. Ser5, and *Eggerthella lenta* significantly increased in D, whereas *Eubacterium rectale*, *Bacteroides fragilis*, *Bacteroides sp*. 1AL, *Finegoldia magna*, *Prevotella pallens*, and *Synergistetes bacterium* SGP1 showed higher abundances in A (Table S2: Mean relative frequency of abundance (%) of phyla, classes, orders, genera, and species of caecal bacteria in 42-day old broilers belonging to groups A and D; Figure S1: Caecal bacteria with > 0.1% mean relative frequency of abundance in A vs. D groups).

## 4. Discussion

Chicken feed additives containing microorganisms (i.e., probiotics and synbiotics) have been commonly administered through feed or drinking water. However, exposing chickens to microbial supplements immediately after hatch can promote an early GI colonization by desirable strains, with positive implications for health and productivity [23,25]. Therefore, our experiment aimed to evaluate how three administration programs of a synbiotic (i.e., sprayed post-hatch as gel droplets onto chicks’ feathering or included in-feed throughout the rearing period, or both) differently affect broilers’ performance and FPD.

Treatment strategies based on post-hatch administration and whole life feeding alone did not show any meaningful result in terms of performance improvement, FPD reduction, and caecal microbiota profiles (Table 2; Table 4; Figure 2). Such outcomes can be attributed to multiple factors influencing probiotics’ action, namely animal age and physiological state, diet, environment, GI microbiota balance, bacterial composition of the additive, processing and stability variations, dose and viability, timing and route of administration, and persistency in the GIT [16,17].

Nevertheless, significant differences in terms of FCR, FPD, and mortality emerged only by comparing, with Student’s *t*-test and Chi-square test, the control (A) with group D that received both synbiotic treatments (Table 3; Table 5). The combination likely had synergistic effects, which were able to overcome the above limitations possibly connected to providing the additive either post-hatch or in-feed. Spraying a CE solution on chicks at the hatchery followed by drinking water administration during growth was tested by Blankenship et al. [42]. The two-step treatment was effective in controlling *Salmonella* infection, but the authors could not evidence whether the double intervention is required to fully counteract such foodborne pathogens. However, few years later Chen et al. [43] demonstrated that combining spray application of a CE preparation onto day-of-hatch broiler chicks with subsequent oral gavaging is indeed more efficacious in lessening intestinal colonization by *Salmonella* than the two single administering routes. Although pursuing different goals compared to the present study, such findings enforce the concept that giving probiotic supplements in more ways and multiple moments can be more beneficial for chicken GI microbiota modulation both from productive and food safety point of view. 

Considering the modulatory potential of synbiotics towards GI microbial communities [44], the significant FCR improvements of group D from 14 to 29 d and in the overall period of the trial (i.e., −4% and −3%, respectively; Table 3) can be attributed to the differences of caecal microbiota compared to the control. According to literature, broiler flocks with different feed to gain ratios are characterized by distinct caecal bacterial assemblages [45,46]. Therefore, it was speculated that the GI microbiota influences chicken productivity and the factors contributing to shape the microbiota may in turn affect chicken performance [47]. Nonetheless, Pan and Yu [48] urged further in-depth analyses in order to establish if differences of GI microbial profile are the cause or the consequence of FCR variations. In addition, a myriad of external and host-related drivers can affect chicken gastroenteric microbiota [49] while experimental design, differences in biological samples (e.g., lumen content or mucosa), sampling procedure and timing, DNA extraction protocol, choice of microbial genome regions to sequence, primers used, and sequencer adopted make comparisons among GI microbiota research arduous [50,51,52,53].

Interestingly, Bäckhed et al. [54] postulated that the microbial composition of the “bioreactor” (as they nicknamed the distal gut) influences the energy-harvesting potential of GI microbiota relative to host’s diet. In the present study significant changes at each taxonomic level were observed between A and D caecal samples (Table S2: Mean relative frequency of abundance (%) of phyla, classes, orders, genera, and species of caecal bacteria in 42-day old broilers belonging to groups A and D). Mean relative frequency of abundance of Actinobacteria and Firmicutes enlarged (0.22% vs. 0.67% and 74.20% vs. 80.27%, respectively), whereas that of Bacteroidetes decreased (22.42% vs. 16.55%) in response to the double synbiotic administration. Therefore, Firmicutes to Bacteroidetes (F/B) ratio of group D was higher than A one. This parameter regarding the microbial hindgut population became a hot topic for the scientific community. Ley et al. [55] stated that shifts of this ratio in favor of Firmicutes could be related to human obesity. Albeit some studies reported no association between F/B ratio and BW of human [56,57,58] or broiler chicken [59], our results support the hypothesis that the higher the F/B ratio, the greater the potential of the intestinal microbiota to collect energy in favor of the host. Obviously, the implications of this theory are substantially diverse for human medicine and broiler husbandry, but such parallelism might offer livestock scientists another piece of knowledge to interpret complex dynamics concerning the host-microbiota interplay.

It should be highlighted that the double treatment led to more beneficial caecal microbial consortium, as indicated by higher abundance of desirable bacterial species coupled with lower concentration of unwanted ones (Table S2: Mean relative frequency of abundance (%) of phyla, classes, orders, genera, and species of caecal bacteria in 42-day old broilers belonging to groups A and D; Figure S1: Caecal bacteria with >0.1% mean relative frequency of abundance in A vs. D groups).

Indeed, *Bifidobacterium longum* and *Collinsella intestinalis* abundances were higher in D birds than A ones. The valuable role of *Bifidobacterium* for livestock is a well-established knowledge [60] while *Collinsella intestinalis* can produce formate and lactate via fermentation processes [61]. The undissociated form of organic acids (e.g., SCFAs and lactate) can penetrate the microbial membrane and dissociate into protons and anions, acidifying cytoplasm. Protons in excess must be removed through an active transport resulting in energy depletion that, coupled with organic acids’ interference with membrane structure and cellular functionality, leads to bacteriostatic or even bactericidal effect against sensitive bacteria [62]. Furthermore, organic acids lower GIT pH favoring the proliferation of desired bacteria (e.g., *Bifidobacterium*) and limiting the growth of harmful ones [63].

D birds showed greater abundance of *Lactobacillus panis*, *Lactobacillus reuteri*, and uncultured *Streptococcus* sp. As exhaustively reviewed in a FAO report [60], a huge number of trials has assessed members of *Lactobacillus* as probiotic candidates intended for livestock, frequently obtaining positive health and performance results. 

Many representatives of Clostridia class significantly increased in response to the twofold synbiotic treatment. *Clostridium difficile*, *Eubacterium ramulus*, *Peptoniphilus asaccharolyticus*, *Ruminococcus obeum, Blautia producta, Blautia* sp. Ser5, along with two exponents of Erysipelotrichi class (i.e., *Clostridium cocleatum* and *Clostridium innocuum*), were more abundant in the synbiotic-fed group. Even if no confirmed advantageous role for the host have been found in literature, especially regarding *Clostridium difficile* [64], most of bacteria previously listed is able to produce useful SCFAs [65,66,67,68,69], which positively affect the GI ecosystem as previously discussed. 

The abundance contraction of *Eubacterium rectale* stands out within Clostridia class (0.14% vs. 0.05%, for A and D respectively). This can be a questionable effect ascribable to the double synbiotic treatment because, at least in humans, such strain, along with the renowned *Faecalibacterium prausnitzii*, is expected to play a crucial role in butyrate production [70]. However, its reduction contextually occurred with the noteworthy increasing of *Eubacterium ramulus* (0.09% vs. 0.35%, for A and D respectively), which is another confirmed butyrate-producer [65]. In this regard, De Cesare et al. [36], evaluating the effects of a probiotic on broilers, explained the growth promotion of some caecal butyrate producers through the alleged cross-feeding mechanism between them and the supplemented *Lactobacillus* strain. The important role of butyrate in GI homeostasis has been widely accepted [71]. Notably, Torok et al. [45] and Stanley et al. [46] associated butyrate-producing caecal bacteria with high performing broilers.

It should be observed that D birds were characterized by significantly lower abundances of *Bacteroides fragilis* and *Bacteroides* sp. 1AL (14.57% vs. 10.12% and 0.11% vs. 0.04%, for A and D respectively). The propionate-producing ability of *Bacteroides* can positively affect human gut health [72,73]. Furthermore, two *Bacteroides* species (i.e., *B. thetaiotaomicron* and *B. vulgatus*) have been recently tested in vitro as components of a possible probiotic preparation intended for restabilising human gut eubiosis after antibiotic treatments [74]. Even though in our experiment the double synbiotic administration did not support *Bacteroides fragilis* and *Bacteroides* sp. 1AL growth, we detected the increase of other organic acids-producer bacteria. Overall, the higher concentration of valuable organic acids in treated caeca could likely explain the significant FCR improvements. Such hypothesis should be confirmed in future studies by means of specific analyses on caecal content chemical composition.

We observed caecal pathogen load reduction since the treatments’ combination significantly decreased *Finegoldia magna*, *Prevotella pallens*, and *Synergistetes bacterium* SGP1. These microorganisms, or at least the genus they belong to, have been associated to infections and severe diseases affecting humans and animals [75,76,77,78,79,80]. However, the growth-promotion of *Eggerthella lenta* (formerly *Eubacterium lentum*), being implicated in numerous illnesses [81], is controversial and difficult to interpret.

FPD decrease can be clarified in the light of supposed intestinal eubiosis prompted by the synbiotic provided from hatch to slaughter. As extensively reviewed by Shepherd and Fairchild [82], poultry FPD is a multifactorial problem, but litter quality and litter management play key roles in its aetiology. Assuming that bedding material and litter management were equal for all the experimental groups, FPD reduction might have been caused either by caecal microbiota modifications or better feed efficiency, both positively affecting litter conditions. 

Lastly, the double treatment did not provoke considerable variation of plasma metabolomes. The significant differences of ascorbic acid and propylene-glycol levels find neither validation nor contradiction in literature, deserving further study.

## 5. Conclusions

Assessing alternative supplementation programs of a synbiotic intended for broilers was the primary aim of this study. Our results suggest favorable effects of the early distribution of synbiotic by spraying gel droplets onto chick feathering at the hatchery, combined with in-feed supplementation during the fattening cycle. Conversely, other administration programs tested in the trial (i.e., only post-hatch or in-feed throughout the growing period) did not determine neither productivity nor health significant variations. The early positive microbial settlement and the lasting favorable microbiota modulation, both supported by the combined treatment, might have been synergistic generating the discrepancy in terms of supplementation programs’ output.

The significant feed efficiency improvements of the group subjected to both synbiotic treatments (i.e., at the hatchery and in-feed) can be presumably ascribed to the modulatory effect of the additive towards caecal microbial community. Such hypothesis could be supported by the significant variations of bacterial taxonomic composition observed in caecal contents of control group and the treated one. The substantial lessening of FPD in the supplemented group might be attributed to possible ameliorations of litter quality guaranteed by better feed efficiency and establishment of favorable gut conditions. The significant differences of plasma ascorbate and propylene-glycol levels produced by the double treatment remain difficult to interpret and should require more investigations.

Overall, our findings confirm the usefulness of synbiotics to improve broiler health and productive performance yet further encouraging researchers to decipher the underlying mechanisms by which such additives influence chicken gastroenteric ecosystem.

## Figures and Tables

**Table 1:** Basal diet composition according to the feeding phase.

Ingredients (g/100 g)	Starter (0–14 d)	Grower (15–28 d)	Finisher (29–42 d)
Corn	42.17	34.96	12.73
White corn	0.00	0.00	15.00
Wheat	10.00	20.00	25.01
Sorghum	0.00	0.00	5.00
Soybean meal	23.11	20.63	17.60
Expanded soybean	10.00	10.00	13.00
Sunflower	3.00	3.00	3.00
Corn gluten meal	4.00	3.00	0.00
Soybean oil	3.08	4.43	5.48
Dicalcium phosphate	1.52	1.20	0.57
Calcium carbonate	0.91	0.65	0.52
Sodium bicarbonate	0.15	0.10	0.15
Salt	0.27	0.27	0.25
Choline chloride	0.10	0.10	0.10
Lysine sulphate	0.59	0.55	0.46
Dl-methionine	0.27	0.29	0.30
Threonine	0.15	0.14	0.14
Xylanase	0.08	0.08	0.08
Phytase	0.10	0.10	0.10
Vitamin-mineral premix ^1^	0.50	0.50	0.50
**Proximate composition (g/100 g)**			
Dry matter	88.57	88.65	88.64
Protein	22.70	21.49	19.74
Lipid	7.06	8.24	9.74
Fiber	3.08	3.04	3.07
Ash	5.85	5.17	4.49
Lys	1.38	1.29	1.21
Ca	0.91	0.80	0.59
P	0.63	0.57	0.46
ME * (kcal/kg)	3076	3168	3264

^1^ Provided the following per kg of diet: vitamin A (retinyl acetate), 13,000 IU; vitamin D3 (cholecalciferol), 4000 IU; vitamin E (DL-α_tocopheryl acetate), 80 IU; vitamin K (menadione sodium bisulfite), 3 mg; riboflavin, 6.0 mg; pantothenic acid, 6.0 mg; niacin, 20 mg; pyridoxine, 2 mg; folic acid, 0.5 mg; biotin, 0.10 mg; thiamine, 2.5 mg; vitamin B12 20 μg; Mn, 100 mg; Zn, 85 mg; Fe, 30 mg; Cu, 10 mg; I, 1.5 mg; Se, 0.2 mg; ethoxyquin, 100 mg.* Metabolizable energy

**Table 2:** Productive performance (mean ± SD) in each feeding phase and in the overall period of the trial *.

Variables	A	B	C	D	*p*-Value
- n.	9	10	9	10	
Starter 0–14 d					
Chick body weight (g/bird)	49.6 ± 0.7	50.1 ± 0.8	49.1 ± 0.9	49.2 ± 0.9	0.07
Body weight (g/bird)	470 ± 38.7	458.1 ± 30.2	472.8 ± 21.4	462.1 ± 28.2	0.70
Daily weight gain (g/bird/d) ^1^	29.8 ± 2.8	29.1 ± 2.1	30.1 ± 1.3	29.5 ± 2.0	0.79
Daily feed intake (g/bird/d) ^1^	39.5 ± 3.9	38.3 ± 2.5	40.4 ± 2.1	39.5 ± 2.5	0.44
Feed intake (kg/bird) ^1^	0.55 ± 0.05	0.54 ± 0.04	0.57 ± 0.03	0.55 ± 0.03	0.44
Feed conversion ratio ^1^	1.325 ± 0.05	1.318 ± 0.11	1.342 ± 0.05	1.340 ± 0.04	0.83
Mortality (%)	2.33 ± 2.22	0.83 ± 1.76	1.39 ± 2.95	0.00	0.10
Grower 15–29 d					
Body weight (g/bird)	1731 ± 61	1737 ± 63	1755 ± 46	1756 ± 35	0.65
Daily weight gain (g/bird/d) ^1^	83.9 ± 3.0	84.7 ± 2.2	85.3 ± 2.3	85.8 ± 1.6	0.32
Daily feed intake (g/bird/d) ^1^	131.7 ± 6.5	128.4 ± 12.5	131.2 ± 2.5	129.5 ± 2.9	0.76
Feed intake (kg/bird) ^1^	1.98 ± 0.10	1.93 ± 0.19	1.97 ± 0.04	1.94 ± 0.04	0.76
Cumulative feed intake (kg/bird) ^1^	2.53 ± 0.14	2.46 ± 0.20	2.53 ± 0.05	2.50 ± 0.06	0.61
Feed conversion ratio ^1^	1.570 ± 0.07	1.519 ± 0.17	1.538 ± 0.04	1.509 ± 0.04	0.53
Cumulative feed conversion ratio ^1^	1.506 ± 0.05	1.467 ± 0.14	1.487 ± 0.04	1.467 ± 0.03	0.67
Mortality (%)	0.00	1.67 ± 2.15	1.39 ± 2.08	0.83 ± 1.76	0.20
Cumulative mortality (%)	2.31 ± 2.20	3.33 ± 1.76	2.78 ± 4.17	0.83 ± 1.76	0.37
Finisher 30–42 d					
Body weight (g/bird)	3175 ± 98	3190 ± 93	3221 ± 95	3242 ± 83	0.40
Daily weight gain (g/bird/d) ^1^	111.5 ± 4.6	110.7 ± 6.8	112.4 ± 4.7	114.2 ± 5.7	0.55
Daily feed intake (g/bird/d) ^1^	201.5 ± 6.6	201.4 ± 5.5	199.8 ± 9.3	200 ± 5	0.92
Feed intake (kg/bird) ^1^	2.62 ± 0.09	2.62 ± 0.07	2.60 ± 0.12	2.60 ± 0.07	0.92
Feed conversion ratio ^1^	1.809 ± 0.07	1.823 ± 0.09	1.780 ± 0.09	1.753 ± 0.06	0.18
Mortality (%)	0.00	1.35 ± 2.16	0.51 ± 1.52	0.87 ± 1.83	0.34
Overall experiment duration 0–42 d					
Chick body weight (g/bird)	49.6 ± 0.7	50.1 ± 0.8	49.1 ± 0.9	49.2 ± 0.9	0.07
Body weight (g/bird)	3175 ± 98	3190 ± 93	3221 ± 95	3242 ± 83	0.40
Daily weight gain (g/bird/d) ^1^	74.3 ± 2.3	74.6 ± 2.2	75.3 ± 2.2	75.9 ± 1.9	0.37
Daily feed intake (g/bird/d) ^1^	120.7 ± 4.0	118.5 ± 4.7	119.3 ± 2.0	119.4 ± 2.2	0.61
Feed intake (kg/bird) ^1^	5.15 ± 0.17	5.08 ± 0.21	5.13 ± 0.14	5.09 ± 0.09	0.79
Feed conversion ratio ^1^	1.643 ± 0.05	1.625 ± 0.09	1.617 ± 0.04	1.596 ± 0.03	0.34
Mortality (%)	2.32 ± 2.2	4.58 ± 2.37	3.24 ± 4.55	1.67 ± 2.15	0.25

* Groups A, B, C, and D; ^1^ Corrected for mortality.

**Table 3:** Productive performance (mean ± SD) in each feeding phase and in the overall period of the trial *.

Variables	A	D	*p-*value
n.	9	10	
Starter 0–14 d			
Chick body weight (g/bird)	49.6 ± 0.7	49.2 ± 0.9	0.57
Body weight (g/bird)	470 ± 38.7	462.1 ± 28.2	0.63
Daily weight gain (g/bird/d) ^1^	29.8 ± 2.8	29.5 ± 2.0	0.77
Daily feed intake (g/bird/d) ^1^	39.5 ± 3.9	39.5 ± 2.5	0.99
Feed intake (kg/bird) ^1^	0.55 ± 0.05	0.55 ± 0.03	0.99
Feed conversion ratio ^1^	1.325 ± 0.05	1.340 ± 0.04	0.44
Mortality (%)	2.33 ± 2.22	0.00	< 0.01
Grower 15–29 d			
Body weight (g/bird)	1731 ± 61	1756 ± 35	0.28
Daily weight gain (g/bird/d) ^1^	83.9 ± 3.0	85.8 ± 1.6	0.09
Daily feed intake (g/bird/d) ^1^	131.7 ± 6.5	129.5 ± 2.9	0.34
Feed intake (kg/bird) ^1^	1.98 ± 0.10	1.94 ± 0.04	0.35
Cumulative feed intake (kg/bird) ^1^	2.53 ± 0.14	2.50 ± 0.06	0.51
Feed conversion ratio ^1^	1.570 ± 0.07	1.509 ± 0.04	0.03
Cumulative feed conversion ratio ^1^	1.506 ± 0.05	1.467 ± 0.03	0.06
Mortality (%)	0.00	0.83 ± 1.76	0.17
Cumulative mortality (%)	2.31 ± 2.20	0.83 ± 1.76	0.29
Finisher 30–42 d			
Body weight (g/bird)	3175 ± 98	3242 ± 83	0.13
Daily weight gain (g/bird/d) ^1^	111.5 ± 4.6	114.2 ± 5.7	0.27
Daily feed intake (g/bird/d) ^1^	201.5 ± 6.6	200 ± 5	0.57
Feed intake (kg/bird) ^1^	2.62 ± 0.09	2.60 ± 0.07	0.57
Feed conversion ratio ^1^	1.809 ± 0.07	1.753 ± 0.06	0.07
Mortality (%)	0.00	0.87 ± 1.83	0.17
Overall experiment duration 0–42 d			
Chick body weight (g/bird)	49.6 ± 0.7	49.2 ± 0.9	0.37
Body weight (g/bird)	3175 ± 98	3242 ± 83	0.13
Daily weight gain (g/bird/d) ^1^	74.3 ± 2.3	75.9 ± 1.9	0.11
Daily feed intake (g/bird/d) ^1^	120.7 ± 4.0	119.4 ± 2.2	0.42
Feed intake (kg/bird) ^1^	5.15 ± 0.17	5.09 ± 0.09	0.41
Feed conversion ratio ^1^	1.643 ± 0.05	1.596 ± 0.03	0.01
Mortality (%)	2.32 ± 2.20	1.67 ± 2.15	0.82

* Groups A and D; ^1^ Corrected for mortality.

**Table 4:** Incidence and severity of FPD at 42 d *.

Variables	A	B	C	D
Number of birds	213	207	206	224
Score 0 (no lesion) (%)	83.1	88.4	92.7	95.1
Score 1 (moderate lesions) (%)	10.8	4.8	4.4	4.0
Score 2 (severe lesions) (%)	6.1	6.8	2.9	0.9
Chi-square test (*p*-value)	0.07			

* Groups A, B, C and D.

**Table 5:** Incidence and severity of FPD at 42 d *.

Variables	A	D
Number of birds	213	224
Score 0 (no lesion) (%)	83.1	95.1
Score 1 (moderate lesions) (%)	10.8	4.0
Score 2 (severe lesions) (%)	6.1	0.9
Chi-square test (*p*-value)	0.02	

* Groups A and D.

## Supplementary Materials

The following are available online at: https://www.mdpi.com/2076-2615/10/3/522/s1. Table S1: Plasma metabolomics profiles according to replication; Table S2: Mean relative frequency of abundance (%) of phyla, classes, orders, genera, and species of caecal bacteria in 42-day old broilers belonging to groups A and D; Figure S1: Caecal bacteria with > 0.1% mean relative frequency of abundance in A vs. D groups.
