# Supplementary material for: Effects of Alternative Administration Programs of a Synbiotic Supplement on Broiler Performance, Foot Pad Dermatitis, Caecal Microbiota, and Blood Metabolites
Source: Animals (Basel). 2020 Mar 20;10(3):522. doi: 10.3390/ani10030522 (PMC7143825; doi:10.3390/ani10030522)

**Supplementary Materials**


**Table S1:** Plasma metabolomics profiles according to replication

Please see the Excel file entitled “Table_S1”.

**Table S2:** Mean relative frequency of abundance (%) of phyla, classes, orders, genera, and species of caecal bacteria in 42-day old broilers belonging to groups A and D.

Please see the Excel file entitled “Table_S2”.

**Figure S1:** Caecal bacteria with > 0.1% mean relative frequency of abundance in A vs. D groups.


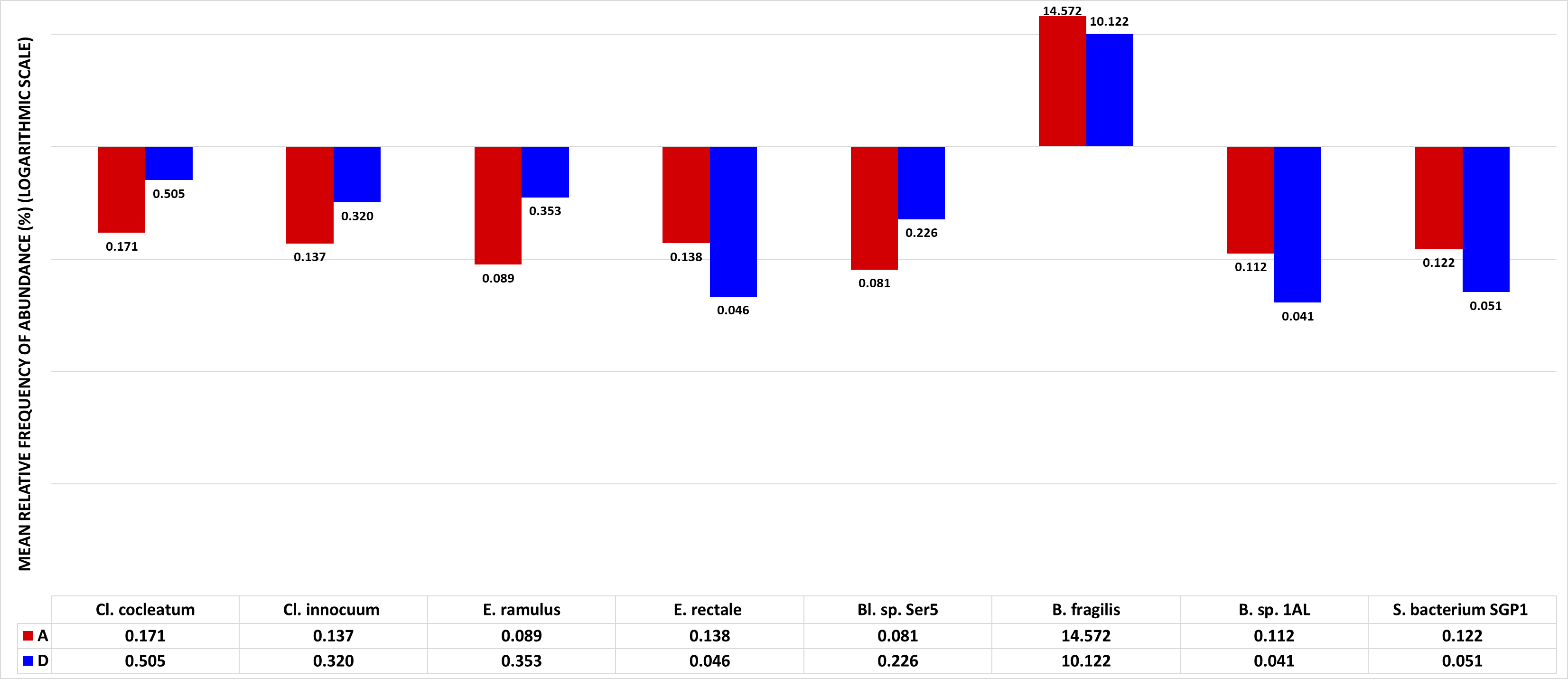

Supplement: Supplementary file 1 [file animals-10-00522-s001.zip › Supplementary/Supplementary_materials R1.docx]
